# Concentrations, Source Characteristics, and Health Risk Assessment of Toxic Heavy Metals in PM_2.5_ in a Plateau City (Kunming) in Southwest China

**DOI:** 10.3390/ijerph182111004

**Published:** 2021-10-20

**Authors:** Xinyu Han, Shuai Li, Zezheng Li, Xiaochen Pang, Yuzhai Bao, Jianwu Shi, Ping Ning

**Affiliations:** 1 Faculty of Civil Engineering and Mechanics, Kunming University of Science and Technology, Kunming 650500, China; 20192110038@stu.kust.edu.cn; 2 Faculty of Environmental Science and Engineering, Kunming University of Science and Technology, Kunming 650500, China; ar19936543210@163.com (Z.L.); 20192207085@stu.kust.edu.cn (X.P.); 20192207012@stu.kust.edu.cn (Y.B.); 11304003@kust.edu.cn (P.N.)

**Keywords:** PM_2.5_, heavy metals, enrichment factor, principal component analysis, heath risk assessment

## Abstract

To explore the mass concentration levels and health risks of heavy metals in the air in dense traffic environments, PM_2.5_ samples were collected at three sites in the city of Kunming in April and October 2013, and January and May 2014. Ten heavy metals––V, Cr, Mn, Co, Ni, Cu, Zn, As, Cd and Pb––were analyzed by ICP–MS, and the results showed PM_2.5_ concentrations significantly higher in spring and winter than in summer and autumn, especially for Zn and Pb. The concentration of heavy metals on working days is significantly higher, indicating that vehicle emissions are significant contributors. An enrichment factor analysis showed that Cr, Mn, Ni, Cu, Zn, As, Cd and Pb come mainly from anthropogenic sources, while V and Co may be both anthropogenic and natural. The correlation and principal component analysis (PCA) showed that Ni, Cu, Zn, Cd and Pb mainly come from vehicles emissions and metallurgical industries; Cr and Mn, from vehicles emissions and road dust; and As, mainly from coal combustion. The health risk assessment shows that the non-carcinogenic risk thresholds of the heavy metals in PM_2.5_ to children and adult men and women are all less than 1. The carcinogenic risk of Cr for men and women in traffic-intensive areas exceeds 10^−4^, reaching 1.64 × 10^−4^ and 1.4 × 10^−4^, respectively.

## 1. Introduction

In recent decades, the number of motor vehicles and total energy consumption have increased in China, as a result of rapid economic development and urbanization. Today, atmospheric particulate matter (PM) is one of the most significant air contaminants [1,2,3]. PM less than 2.5 µm (PM_2.5_) comprises heavy metals, bacteria, toxins and carcinogens due to its small particle size and large specific surface area [4,5]; consequently, it has received extensive attention from researchers. Studies have shown the adverse health effects from exposure to PM and heavy metals [6,7,8,9], and epidemiological studies over the past few decades identified a close relationship between increased atmospheric heavy metals and increased mortality and morbidity [10,11,12].

The heavy metals come from natural and anthropogenic sources, including coal combustion, traffic emissions, and smelting. Because of their high toxicity, persistence and bioaccumulation, research into the health effects of long or short-term exposure to traffic-related air pollutants has become the focus of attention [13,14,15,16]. Fang et al. [17] proposed that heavy metals in road dust are not easy to remove, and may diffuse into the atmosphere and persist in the environment for a long time. He et al. [18] believe that large and medium-sized cities show obvious effects of vehicle pollution, and a large number of studies found high concentrations of Cr, Cu, Zn, Cd and Pb. These metals can cause respiratory and cardiovascular disease, and increase the risk of cancer [19,20,21,22,23,24]. Recent studies also showed that exposure to As, Cd, Hg and Pb in early or late pregnancy is associated with childhood asthma [25].

According to statistics from 2014 to 2019, deaths from respiratory diseases in Yunnan Province were 115.26 per 100,000 people, much higher than the national rate of 69.47/100,000 [26,27,28,29]. We conducted this study to find out whether heavy metals of PM_2.5_ had an impact on the mortality rate of people in Yunnan. However, most atmospheric pollution studies in China focus on the Beijing–Tianjin–Hebei region and coastal areas [30,31]; only a few have investigated PM_2.5_ pollution in Yunnan, and data on heavy-metal pollution is lacking. Therefore, we collected PM_2.5_ samples at three sites in Kunming from 2013 and 2014 to explore the contribution of heavy metals to environmental pollution and human health.

Kunming is in the middle of the Yunnan–Guizhou Plateau in southwestern China (24°23′–26°22′ N, 102°10′–103°40′ E) and the capital of Yunnan Province. Its population is 6.95 million, one of the largest cities on the plateau. Kunming is to the northeast of the Dianchi Basin and surrounded by mountains on three sides, with an average altitude of 1891 m. The overall terrain gradually decreases from north to south in a step-like manner. Kunming has a subtropical–plateau mountain monsoon climate and the annual average urban temperature is about 15 °C. The year-round temperature difference is small, but the daily temperature varies greatly, and the ultraviolet intensity is high, which means inversion layers form easily, hindering the diffusion of pollutants. The mountainous regions of the Yunnan–Guizhou Plateau are rich in minerals, and the densely distributed non-ferrous metallurgical enterprises around Kunming City are a source of atmospheric heavy metal pollution. In addition, Kunming is the most important transportation hub in Yunnan Province. As the economy opened up to Southeast Asia car ownership rose to 1.8 million in 2013, and in 2019 it was 58.29% higher [32,33]. Traffic density in Kunming in 2013 was 1570 vehicles/km^2^, comparable to that of Beijing (2030) [34], Chengdu (1320) [35], and Zhengzhou (2070) [36].

Because industries have moved to industrial parks away from urban areas, motor vehicle PM emissions seem to be the critical source of pollutants. Therefore, we conducted field measurements of the seasonal variations of PM_2.5_ heavy metals in Kunming to quantify the contribution of various sources, particularly motor vehicles, and assess the associated health risks. This study provides essential information for developing control strategies and protecting the health of people on the Yunnan–Guizhou Plateau.

## 2. Materials and Methods

### 2.1. Sampling Sites Description

**Figure 1:**
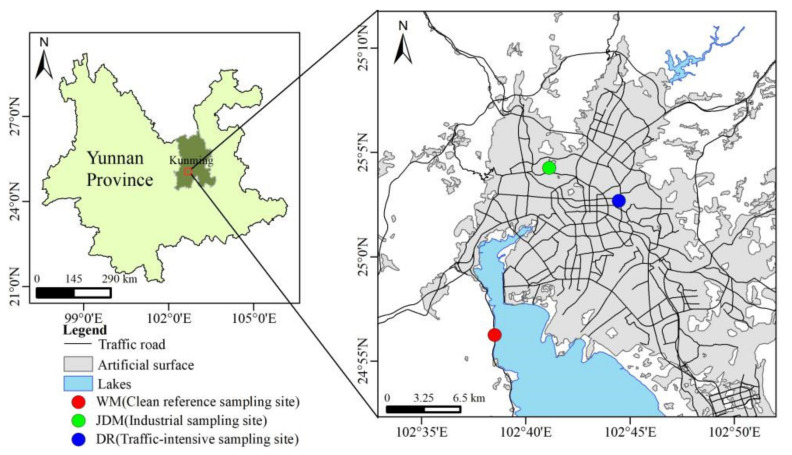
Location map of the sampling sites in Kunming (WM: West Mountain, Clean reference sampling site; JDM: Jinding Mountain, Industrial sampling site; DR: Dongfeng East Road, Traffic-intensive sampling site; km: kilometer; N in the upper left corner of the picture: North arrow; N on the ordinate in the figure: North latitude; E on the abscissa in the figure: East longitude).

The sampling of PM_2.5_ was performed at three sites in Kunming (Figure 1). Dongfeng East Road (DR, 25°24′ N, 102°42′ E; 1900 m AMSL), near a main road with a traffic volume of 3436 vehicles·h^−1^; Jinding Mountain (JDM, 25°0′ N, 102°41′ E; 1920 m AMSL), which is near steel and glass industries and where traffic volume is about 562 vehicles·h^−1^; and West Mountain (WM, 24°57′ N, 102°37′ E; 2300 m AMSL), a clean reference.

### 2.2. Sampling Schedule and Method

PM_2.5_ samples for 28 days were collected during 19–25 April and 24–30 October 2013, and 9–15 January and 21–28 May 2014. Daily samples were collected simultaneously at the sites for 23 ± 1 h from 9:00 a.m. to 9:00 a.m. During the sampling period, three standard medium-volume PM_2.5_ samplers (Wuhan Tianhong Intelligent Instrument, Model TH150) were employed at a flow rate of 100 L·min^−1^. Before sampling, we calibrated the flow rate with a soap film flowmeter (Qingdao Laoshan application technology research institute, Model 7030). Airborne particles were collected on 90 mm-diameter polypropylene-fiber filters, which had been annealed at 75 °C for 0.5 h to remove any impurities. The filters were stored in a desiccator at 25 °C and 45% relative humidity for 48 h before and after sampling and then weighted, packed in sample cells and stored at 15–30 °C till extraction and analysis. All filters were analyzed within 2 weeks. Table 1 lists the detailed sampling time and weather conditions at the three sites. Wind speed and temperature data are derived from https://www.wunderground.com/ (accessed on 23 August 2021).

### 2.3. Heavy Metal Analysis

Inductively Coupled Plasma Mass Spectrometry (ICP–MS, Agilent 7500a, Agilent, Palo Alto, CA, USA) analysis was employed to determine concentrations of 10 kinds of heavy metal elements: V, Cr, Mn, Co, Ni, Cu, Zn, As, Cd and Pb. Aluminum (Al) was analyzed to calculate the enrichment factors as the reference element. The scrap sampled filters were immersed in mixture of HCl and HNO_3_ (HCl: 16.7 mL, HNO_3_: 5.5 mL, ultrapure water: 150 mL) in a Teflon beaker. Then the beaker was covered by a lid, and the solution was placed on a 220 °C temperature-controlled electric hot plate for 2.5 h of circumfluence. As the liquid cooled, we washed out the inner wall of the beaker with ultrapure water, stewing for 0.5 h for extraction, filtration, then at constant volume to 50.0 mL under test.

Blanks (including filters) and duplicate sample analyses were performed for approximately 10% of all the samples. Certified reference materials (CRM) were used to ensure accuracy and precision (National Research Center of CRM, China). The field blank samples were analyzed using these same procedures. The results of the blank analyses were used to correct the corresponding samples.

### 2.4. Method

#### 2.4.1. Enrichment Factor (EF)

*EF* is an important indicator of the extent of disturbance to the natural environment caused by human activity. By comparing measured values with soil background values, we can see the influence of human activity on particulates [37]. *EF* is calculated by the following equation:

*EF* = (*C_i_*/*C_n_*)*_sample_*/(*C_i_*/*C_n_*)*_soil background_* (1)

where *C_i_* represents the concentration of heavy metals; *C_n_* represents the concentration of the reference element (we choose Al as the reference element in this study); *(C_i_/C_n_)_sample_* is the concentration ratio of the samples; *(C_i_/C_n_)_soil background_* is the concentration ratio of the corresponding element in the crust. If *EF* is close to 1, the element can be considered to originate mainly from soil particles; if *EF* > 10, the element mainly originates from human activity [38].

#### 2.4.2. Principal Component Analysis (PCA)

PCA is a resource allocation method approved and recommended by the U.S. Environmental Protection Agency (EPA). It is an important multivariate statistical tool that can reduce the dimensionality of large datasets and extract the number of principal components needed to explain all the variances of such datasets, which is much less than the original number of variables [39,40]. This method establishes the orthogonal distribution between the components. No matter how many variables were included in this study [41], the regression adjustment results for each factor were simple and stable. During the analysis, the factors were determined by selecting principal components with eigenvalues greater than 1 according to previous studies [42,43]. They effectively explained and represented the source characteristics of each heavy metal in PM_2.5_, guided the understanding and control of heavy metal pollution emissions in the study area, and helped improve the health of local populations.

#### 2.4.3. Health Risk Assessment (HRA)

This research used the health risk assessment model provided by the U.S. EPA as the basic framework, which was used for assessing the health risk of heavy metals in PM_2.5_ [44]. The target population of this study was children, and adult men and women. Then, based on the concentration levels of heavy metals in the study area, we calculated and assessed the health effects according to the health risk assessment model [45,46]. Because the heavy metals in PM_2.5_ enter the body through the respiratory system, this study focused on assessing the health risks of inhalation exposure, and the dose of this route was calculated by the following formula:

*ADD*(*LADD*)*_Inhale_* = (*C* × *IR_i_* × *EF* × *ED* × *CF*)/(*BW* × *AT*) (2)

where *C* (ng·m^−3^) represents the concentration of heavy metal elements in PM_2.5_; the variable *ADD* (mg·(kg·d)^−1^) is normal average daily dose for non-carcinogenic elements; and *LADD* (mg·(kg·d)^−1^) is lifetime average daily dose for carcinogenic elements. The exposure parameters are mainly based on the exposure parameter manual of the population in China [47] as shown in Table 2.

According to the EPA’s Integrated Risk Information System (IRIS) and the International Agency for Research on Cancer (IARC), pollutants can be divided into carcinogens and non-carcinogens [48]. In this study, V, Mn, Cu, Zn and Pb are non-carcinogens; Cr, Co, Ni, As and Cd are carcinogens. The non-carcinogenic risk quotient (*HQ*) and carcinogenic risk (*CR*) of heavy metals were calculated as follows:

*HQ* = *ADD*/*Rfd* (3)

*CR* = *LADD* × *SF* (4)

In the formula, *Rfd* (mg·(kg·d)^−1^) is the reference dose of each heavy metal; *SF* ((kg·d)·mg^−1^) is the carcinogenic slope factor. The specific parameters were shown in Table 3 [49,50,51]. If *HQ* were less than 1, it meant that the non-carcinogenic risk was small or negligible; if it were greater than or equal to 1, it meant that there was a non-carcinogenic risk, which increased as the *HQ* value increased; *CR* represents the carcinogenic risk of heavy metals: when *CR* was between 10^−6^ and 10^−4^, it was within the acceptable range. If *CR* ≥ 10^−4^, there was a significant risk [52].

#### 2.4.4. HYSPLIT4 Model

The HYSPLIT4 model is a professional model jointly developed by the National Oceanic and Atmospheric Administration (NOAA) Air Resources Laboratory (ARL) and the Australian Bureau of Meteorology (BOM) over the past 20 years to calculate and analyze the transport and diffusion of atmospheric pollutants. The model has a relatively complete transport, diffusion and sedimentation model that can handle multiple meteorological element input fields and physical processes, and different types of pollutant emission sources. It is widely used in the study of the transmission and diffusion of multiple pollutants in various regions. In this study, the independent version of the backward trajectory model and the auxiliary software package were used (GUI; Ghostscript; ImageMagick). Meteorological data were obtained through NCEP (National Centers for Environmental Prediction) and GDAS (Global Data Assimilation System).

## 3. Results and Discussion

### 3.1. Concentrations of Heavy Metals in PM_2.5_ in Kunming

#### 3.1.1. Concentration Variation for Heavy Metals in PM_2.5_ on Working Days and Rest Days

**Figure 2:**
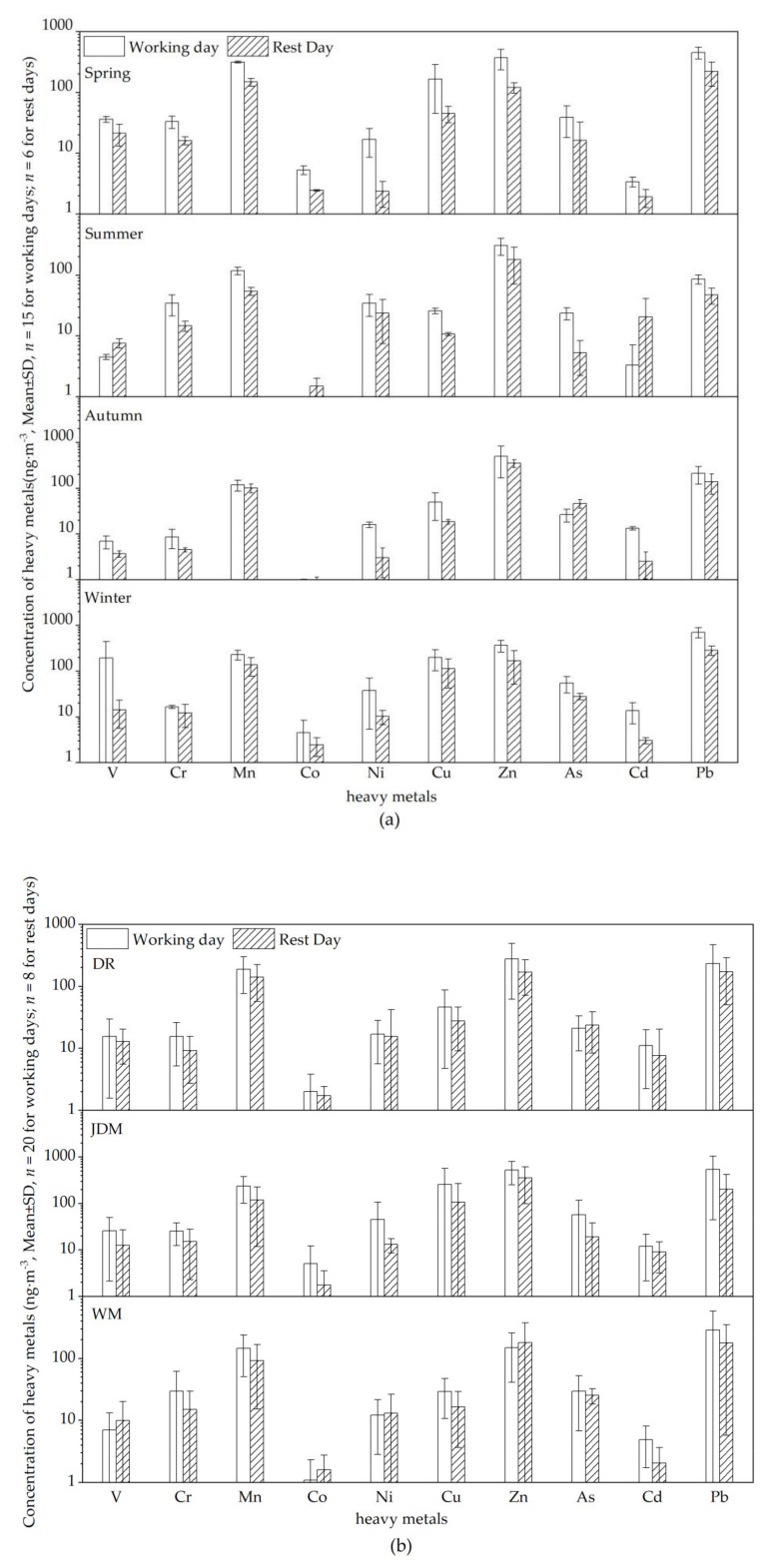
(**a**) Variation in heavy metal concentrations on working and rest days with the seasons; (**b**) Variation of heavy metals concentration on working and rest days at different sampling sites. (V: Vanadium; Cr: Chromium; Mn: Manganese; Co: Cobalt; Ni: Nickel; Cu: Copper; Zn: Zinc; As: Arsenic; Cd: Cadmium; Pb: Lead; WM: West Mountain, Clean reference sampling site; JDM: Jinding Mountain, Industrial sampling site; DR: Dongfeng East Road, Traffic-intensive sampling site; ng·m^−3^: Concentration unit, indicating the number of nanograms per cubic meter; SD: Standard deviation; *n*: Number of samples).

During the four sampling campaigns, the average mass concentrations of PM_2.5_ at the three Kunming sites (DR, JDM, WM) were 125.16 ± 71.27 μg·m^−3^, 170.10 ± 104.10 μg·m^−3^ and 114.00 ± 73.99 μg·m^−3^, respectively. The overall concentration of the 10 heavy metals accounted for about 1.26% of PM_2.5_. Pb and Zn accounted for the highest proportions, 0.32 and 0.38%, respectively. Because most large industries in Kunming had moved to industrial parks far from urban areas, only a few industries were located around the JDM site. These industries were in a continuous operation, which shows that the concentrations of discharged heavy metals may not have fluctuated much between working days and rest days. Heavy-duty diesel vehicles are common around the site and emit a large amount of PM_2.5_ heavy metals on workdays. Figure 2a shows the concentration variation of 10 heavy metals on working days and rest days. The total concentrations ranged from 481.70 to 2052.65 ng·m^−3^ on working days, and 199.97 to 1105.21 ng·m^−3^ on rest days. The concentration levels of heavy metal markers (Cr, Mn, Cu, Zn, and Pb) from traffic sources are significantly higher on working days, especially in spring and winter. Figure 2b shows the concentration variation of heavy metals at the three sampling sites. It was found that almost all of the concentrations fluctuated wildly on working and rest days, especially at the DR and JDM sites, which suggest that heavy metals emitted by the traffic sources make an important contribution to ambient air.

#### 3.1.2. Seasonal and Spatial Variations of Heavy Metals in Kunming

**Figure 3:**
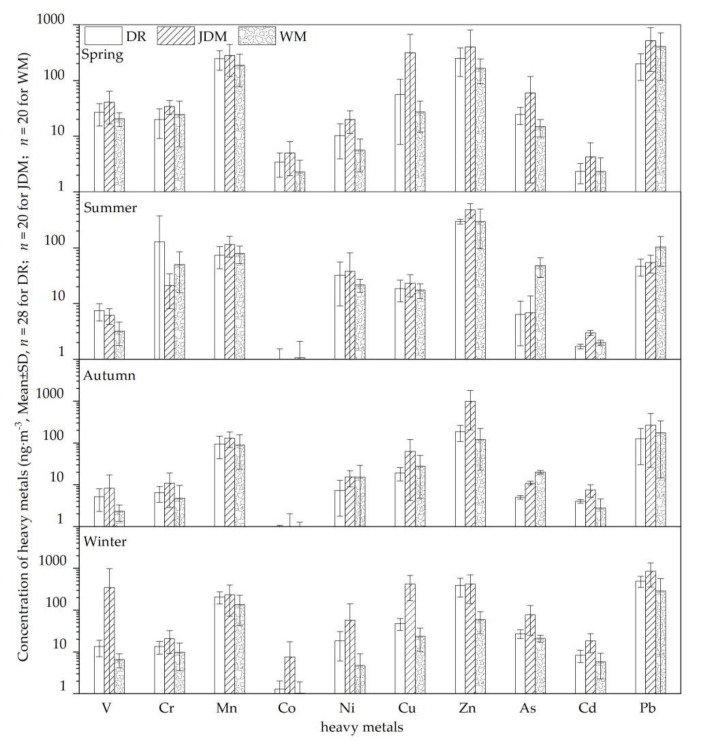
Variation of heavy metals concentrations in three sites of Kunming (V: Vanadium; Cr: Chromium; Mn: Manganese; Co: Cobalt; Ni: Nickel; Cu: Copper; Zn: Zinc; As: Arsenic; Cd: Cadmium; Pb: Lead; WM: West Mountain, Clean reference sampling site; JDM: Jinding Mountain, Industrial sampling site; DR: Dongfeng East Road, Traffic-intensive sampling site; ng·m^−3^: Concentration unit, indicating the number of nanograms per cubic meter; SD: Standard deviation; *n*: Number of samples).

Seasonal and spatial variations in PM_2.5_ heavy metals in Kunming are shown in Figure 3, which also shows the concentration levels of all 10 heavy metals and their standard variance. The concentrations followed the order Zn > Pb > Mn > As > Cu > V > Cr > Ni > Cd > Co. The concentration of Cr, As and Cd exceeded the secondary level of National Ambient Air Quality Standard of China (GB 3095-2012, the limit concentrations of As, Cr and Cd are 6.00, 0.025 and 5.00 ng·m^−3^). The highest concentration was found in autumn at JDM with 996.6 ± 791.56 ng·m^−3^ (Zn), and the lowest concentration was found in autumn at WM with 0.52 ± 0.75 ng·m^−3^ (Co). Overall, the concentrations of Zn and Pb were much higher than other heavy metals, and the concentrations of most were consistent with the seasonal distribution of PM_2.5_, showing a pattern of high concentrations in winter and spring, and low concentrations in summer and autumn. Due to the intensive traffic flow and frequent industrial activity at the two sampling sites DR and JDM, concentrations of Ni, Cu, Zn, Cd and Pb were much higher than that at WM. Concentrations of Cr reached the highest value at DR because of heavy summer traffic (147.80 ± 247.85 ng·m^−3^), but summer concentrations of As and Pb were significantly higher at WM than at DR and JDM, possibly because WM is downwind from the petroleum, steel, phosphate, and salt industries at the Anning Industrial Park. However, there are many high mountains around the WM site that block the transmission of pollutants to Kunming. Therefore, the pollutants from Anning Industrial Park may not have had a significant impact on the DR and JDM sampling sites.

#### 3.1.3. The Transport of Heavy Metals

**Figure 4:**
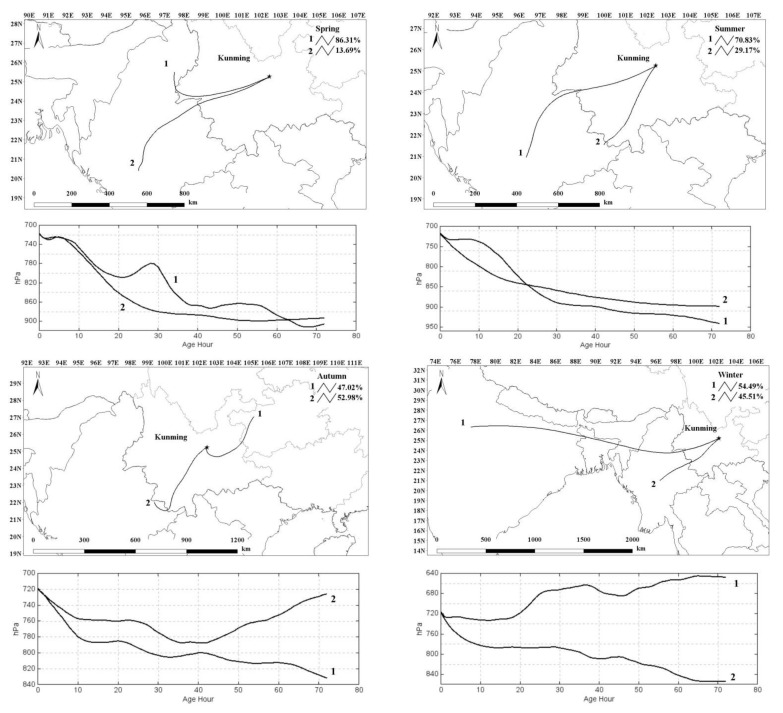
The clustering of air mass in Kunming during the four sampling periods (hPa: Hectopascal; Age Hour: Indicates 72 h before the selected time point of the backward trajectory; N on the upper left corner of the figure: North arrow; N on the left axis: North latitude; E on the upper axis: East longitude; 1 and 2 represent the clustering lines of the air mass trajectories).

To understand the transport of heavy metals in PM_2.5_ from distant sources, 72 h backward trajectories starting at 1000 m at the center of Kunming during the four sampling periods were calculated every 6 h using the Hybrid Single Particle Lagrangian Integrated Trajectory 4.0 (HYSPLIT4) model. Meteorological data came from the Global Data Assimilation System (GDAS). Figure 4 shows the clustering of air mass trajectories at 1000 m in Kunming. It was found that the air masses mainly originated in Myanmar in the southwest during the spring, summer and winter, and 47.02% of autumn air masses originated in Guizhou.

**Figure 5:**
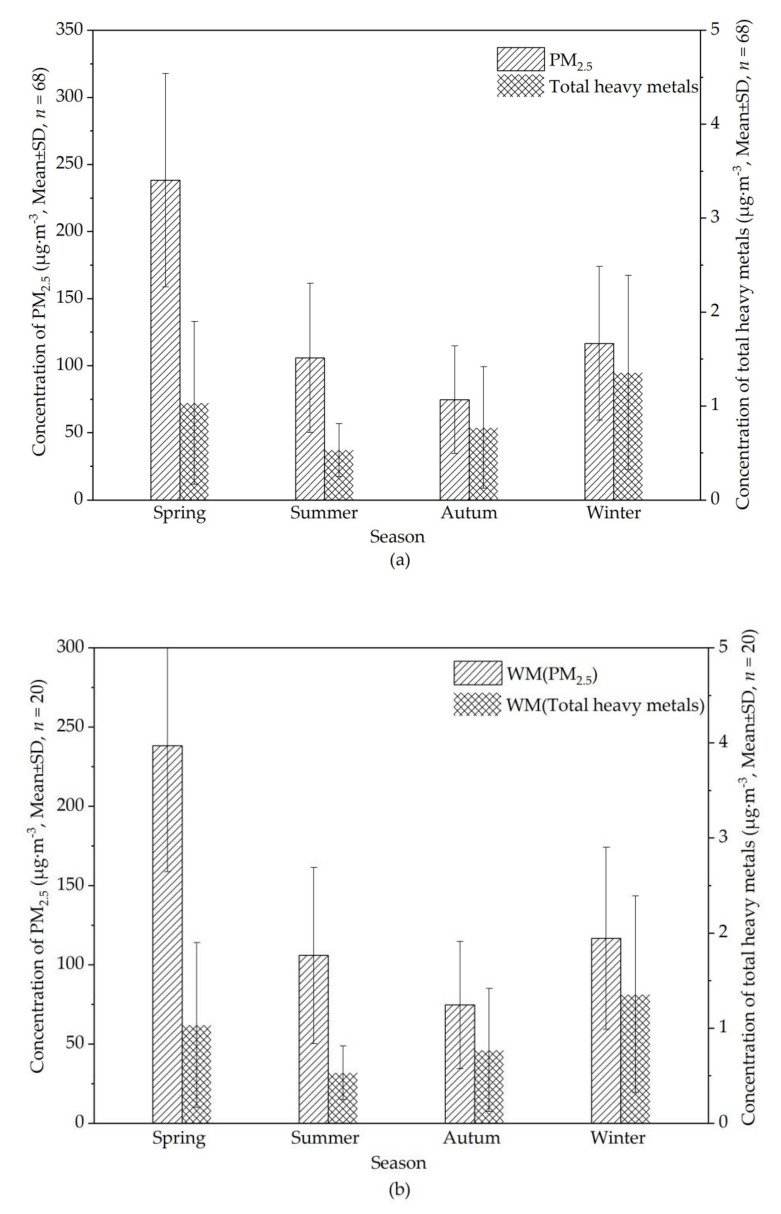
Concentration variation of total heavy metals and PM_2.5_ (**a**) in Kunming and (**b**) at the WM site during all sampling periods (WM: West Mountain, Clean reference sampling site; μg·m^−^^3^: Concentration unit, indicating the number of micrograms per cubic meter; SD: Standard deviation; *n*: Number of samples).

Figure 5 shows the concentration variations of total heavy metals and PM_2.5_ in Kunming (Figure 5a) and at WM (Figure 5b) during the four sampling periods. Combined with Figure 4, we can see that high concentrations occur in spring, possibly caused by the long-distance transmission of dense biomass burning from Myanmar. In summer, due to rainfall, concentrations were relatively low, and in autumn, clean air from Guizhou did not increase concentrations. In winter, however, concentrations of total heavy metals were the highest, which may be the result of coal burning by local and regional residents or of the long-distance transmission of pollution from India.

Figure 5b shows that the concentration variation of total heavy metals and PM_2.5_ at WM during all sampling periods followed the order spring > summer > winter > autumn. In summer, the high concentrations of PM_2.5_ may have been caused by forest fires in the region of Anning during early sampling, or the valley wind that brought pollution up to the WM site on the top of the mountain.

#### 3.1.4. Comparisons with the Concentration of Heavy Metals in Other Cities

As shown in Table 4, the concentrations of most heavy metals in PM_2.5_ were generally higher than for most other cities, but they were lower than in Xi’an [53], which is an important industrial base and comprehensive transportation hub in Northwest China. The concentrations of some carcinogenic heavy metals (Cr, Ni and As) were comparable to those reported in other cities. Kunming’s As and Pb levels were 2.68 and 1.88 times higher, respectively, than those of Beijing [54]. Nanjing, Chengdu, Chongqing and Guangzhou [55,56,57] are all provincial capitals or municipalities with a dense transportation network, but the concentrations of heavy metals are much lower than for Kunming. Compared with Barcelona [58], all heavy metal concentrations for Kunming were higher: Cu, Zn and Pb were more than twice as high and Cr was 4.85 times higher. In Istanbul [59], the level of Cr was 4.18 times higher, but other heavy metals were lower. Similarly, as a plateau mountain city, the city of Lanzhou [60] also has very high concentrations of heavy metals in PM_2.5_. These results may explain to a certain extent the reason why the death rate due to problems with the respiratory system in Yunnan is higher than the national average. However, some cities have higher concentrations of heavy metals and lower death rates, which implies that there are other atmospheric pollutants in the environment that affect the respiratory system, such as persistent organic matter, etc.

### 3.2. Sources of Heavy Metals

#### 3.2.1. Enrichment Factor (EF) and Correlation Analysis

**Figure 6:**
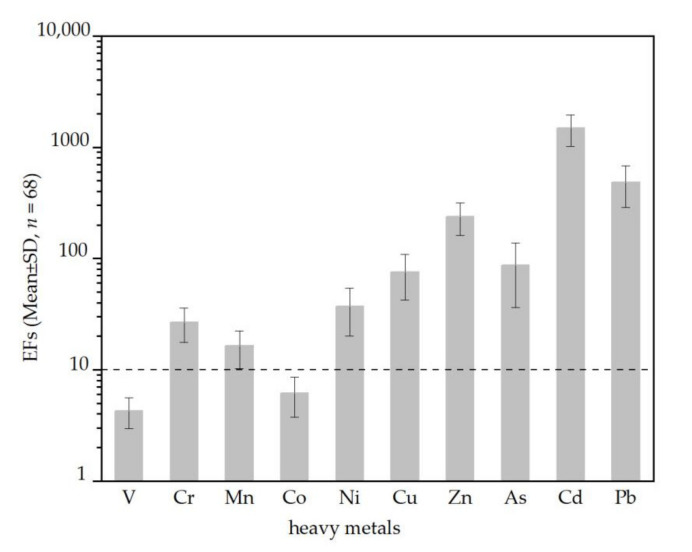
Enrichment factors of heavy metals in PM_2.5_ in Kunming (EFs: Enrichment factors; SD: Standard deviation; *n*: Number of samples; V: Vanadium; Cr: Chromium; Mn: Manganese; Co: Cobalt; Ni: Nickel; Cu: Copper; Zn: Zinc; As: Arsenic; Cd: Cadmium; Pb: Lead).

In this study we used an enrichment factor (EF) and PCA to analyze possible sources of the 10 heavy metals in PM_2.5_ in Kunming. Figure 6 shows that the EFs of Cr, Mn, Ni, Cu, Zn, As, Cd and Pb were higher than 10, which suggested that these heavy metals were significantly affected by anthropogenic sources. The EFs of V and Co were lower than 10 but higher than 1, which suggests that the two elements may have been affected by natural and anthropogenic sources.

The correlation among the elements can indicate the sources of the heavy metals. In this paper, we used SPSS17 software to calculate the correlation coefficient between elements. The coefficient matrix is shown in Table 5.

Table 5 shows that Cr and Mn had the highest correlation coefficient of 0.915. Previous studies showed that Cr, Mn and Ni were the main pollutants in road dust [61,62]. Manoli et al. [63] also reported that road dust had high levels of Cr and Mn, indicating that this might be the source.

V, Ni, Cu and Pb were strongly correlated, and some studies showed that they are usually related to the metallurgical industries and vehicle exhaust emissions [64,65,66]. Cu and Pb may also come from brake and tire wear [67]. V may be influenced by the type of pavement or industrial dust [64,68]. Therefore, V, Ni, Cu and Pb may mainly come from vehicle emissions and metallurgical industries.

There was no significant correlation between As and the other heavy metals since it was commonly used as a coal combustion indicator [69]. The table shows that As in Kunming PM_2.5_ came mainly from coal combustion and did not highly correlate with vehicle emissions.

#### 3.2.2. PCA of Heavy Metals

We use PCA to analyze heavy metal concentration data to identify possible sources of heavy metals in Kunming. The heavy metals data of all samples were input to the PCA model and analysis was performed. As shown in Table 6, three factors loading for heavy metals from PCA were listed, with varimax rotation of the data matrix. Factors 1–3 explained 49.43%, 18.73% and 12.61% of the total variance in the data set, respectively. In Factor 1, the heavy metals Ni, Cu, Zn, Cd and Pb have higher scores, which are 0.892, 0.862, 0.724, 0.912 and 0.880, respectively. The high load values of Ni, Cu, Zn, Cd and Pb indicate that Factor 1 is mainly from vehicle emissions and metallurgical industry sources [70,71]. The heavy metals Cr, Mn and Zn in Factor 2 are markers of traffic and road dust emission sources, so it is inferred that Factor 2 represents vehicle emissions and road dust [72]. In the Factor 3, heavy metal As has the highest score of 0.995. As is a marker component of coal, so it is inferred that Factor 3 is from coal combustion sources [48,73]. According to these results, we can know that vehicle emissions and metallurgical industry sources make the biggest important contribution to heavy metals in Kunming.

### 3.3. Health Risk Assessment of Heavy Metal Elements Exposure

In Kunming, the non-carcinogenic risk of heavy metals in PM_2.5_ at three sampling sites was analyzed through respiration. The results during the entire sampling period are shown in Table 7. Among the three sampling sites, JDM had the highest non-carcinogenic risk of heavy metals, followed by DR and WM, but the trends for all three were the same. The risk levels of non-carcinogenic heavy metals for children, and men and women were Mn > Pb > Cu > V > Zn; and the order is children > men > women. The non-carcinogenic risk for each heavy metal was less than 1, indicating that it was within the acceptable range of respiratory exposure. However, most were affected by Mn and Pb.

The carcinogenic risk of each heavy metal to each groups through respiration is shown in Table 8. At the three sampling sites, the carcinogenic risk of heavy metals for children was DR > WM > JDM; the carcinogenic risk of heavy metals for adults was DR > JDM > WM. The risk levels of carcinogenic heavy metals were Cr > As > Co > Cd > Ni. The carcinogenic risk of Cr to men and women in the DR sampling site exceeded 10^−4^. The carcinogenic risk of other heavy metals in all populations at the three sampling sites was between 10^−6^ and 10^−4^, which was within the acceptable range. Cr posed the highest carcinogenic risk to all people through respiration, and it should receive more attention.

### 3.4. Discussion

We conducted this study to find out whether heavy metals from PM_2.5_ had an impact on the death rate of the respiratory system in Yunnan. In the past few years, the number of deaths due to respiratory diseases in Yunnan Province is much higher than for the whole country, which implies that the environmental pollution has a significant impact on people in Yunnan. Heavy metals emitted from transportation sources may cause harm to the health of urban populations. Car ownership in Kunming has risen sharply in recent years, which is comparable to that of other medium and large cities in China. Therefore, we collected PM_2.5_ samples in Kunming to explore the contribution of heavy metals to environmental pollution and human health. The results are in line with the original assumptions. First, we analyzed the spatial and temporal distribution characteristics of heavy metal concentrations, and found that at sampling site where traffic source emissions are more obvious, the changes in heavy metal concentrations between working days and rest days fluctuate significantly. At the same time, we used PCA to analyze the source of heavy metals. The results show that Cr, Mn, Cu, Zn and Pb are all related to traffic source emissions. In the health risk assessment of heavy metal exposure, Cr has a significant carcinogenic risk for people in Kunming, which may explain to a certain extent the reason why the death rate of the respiratory system in Yunnan is higher than the national average.

There are some limitations to this study. Firstly, there is inadequate explanation regarding the death rate due to problems with the respiratory system in Yunnan, indicating that more variables need considering, for example, the level of persistent organics in the atmosphere. Secondly, the data is a little old, so health risks may not reflect current reality well. Thirdly, due to the complexity of the terrain of the plateau city, we only studied the characteristics of heavy metal pollution in Kunming, which is far from being able to clarify the entire mechanism of atmospheric heavy metal pollution in the plateau area.

## 4. Conclusions

The concentrations of PM_2.5_ at three sites (DR, JDM, WM) were 125.16 ± 71.27, 170.10 ± 104.10 and 114.00 ± 73.99 μg·m^−3^, respectively, and as for the concentrations of the 10 heavy metals (Zn > Pb > Mn > As > Cu > V > Cr > Ni > Cd > Co), Cr, As and Cd exceeded the second level of China’s national standard for ambient air quality (GB 3095-2012).

An EF analysis showed that Cr, Mn, Ni, Cu, Zn, As, Cd and Pb came mainly from anthropogenic sources, and that V and Co may have come from a mixture of natural and anthropogenic sources. For external sources, the clustering of air masses in Kunming showed that they mainly originated in Myanmar during the sampling periods, and the long-distance transmission of dense biomass burning in Myanmar in the spring affected the concentration levels of heavy metals.

We analyzed the concentration of heavy metals on working and rest days. Almost all concentrations fluctuated intensely on working and rest days at all three sampling sites, especially DR and JDM, which suggested that heavy metals from traffic made an important contribution to the ambient air. At the same time, the PCA results show that Ni, Cu, Zn, Cd and Pb came mainly from vehicle and metallurgical industry emissions (49.43% of the total variance), and Cr, Mn and Zn came from vehicle emissions and road dust (18.73% of the total variance). These results all showed that motor vehicles were already a critical air pollution source of heavy metals.

The total non-carcinogenic risk of heavy metals was less than 1, which was in the acceptable range of respiratory exposure. The risk levels of carcinogenic heavy metals were in the order: Cr > As > Co > Cd > Ni. The carcinogenic risk of Cr to men and women at the DR sampling site was greater than 10^−4^.

To reduce urban traffic emissions and protect people’s health, it is recommended that preferential policies be enacted to encourage consumers to use clean energy vehicles on the Yunnan–Guizhou Plateau.

## Figures and Tables

**Table 1:** Sampling time and weather conditions at three sampling sites in Kunming.

Season	Sampling Time	Sampling Sites			Weather	Wind Speed (m·s^−1^)	Concentration of PM_2.5_ (μg·m^−^^3^, Mean ± SD, *n* = 3)	Air Temperature (°C)
		WM	JDM	DR				
Spring	4/18/2013-4/19/2013, (23 ± 1 h)	Y	Y	Y	Cloudy	7.25	162.32 ± 46.36	18.00
	4/19/2013-4/20/2013, (23 ± 1 h)	Y	Y	Y	Cloudy	7.77	267.42 ± 127.15	18.00
	4/20/2013-4/21/2013, (23 ± 1 h)	Y	Y	Y	Clear	4.78	242.5 6± 64.67	20.50
	4/21/2013-4/22/2013, (23 ± 1 h)	Y	Y	Y	Clear	6.67	244.74 ± 41.35	20.50
	4/22/2013-4/23/2013, (23 ± 1 h)	Y	Y	Y	Clear	8.01	293.83 ± 32.78	20.50
	4/23/2013-4/24/2013, (23 ± 1 h)	N	N	Y	Clear	6.71	232.44	21.00
	4/24/2013-4/25/2013, (23 ± 1 h)	N	N	Y	Cloudy	6.82	187.80	21.00
Summer	5/21/2014-5/22/2014, (23 ± 1 h)	Y	Y	Y	Shower	8.72	101.02 ± 4.00	22.50
	5/22/2014-5/23/2014, (23 ± 1 h)	Y	Y	Y	Shower	7.38	117.67 ± 46.90	21.50
	5/23/2014-5/24/2014, (23 ± 1 h)	Y	Y	Y	Shower	7.29	158.44 ± 84.00	22.00
	5/24/2014-5/25/2014, (23 ± 1 h)	Y	Y	Y	Cloudy	6.80	109.13 ± 51.28	21.50
	5/25/2014-5/26/2014, (23 ± 1 h)	Y	Y	Y	Cloudy	7.06	79.69 ± 8.13	22.00
	5/26/2014-5/27/2014, (23 ± 1 h)	N	N	Y	Shower	2.95	53.62	22.00
	5/27/2014-5/28/2014, (23 ± 1 h)	N	N	Y	Shower	4.25	50.65	21.50
Autumn	10/23/2013-10/24/2013, (23 ± 1 h)	Y	Y	Y	Moderate rain	5.36	47.49 ± 17.41	13.50
	10/24/2013-10/25/2013, (23 ± 1 h)	Y	Y	Y	Light rain	2.91	85.37 ± 35.01	13.50
	10/25/2013-10/26/2013, (23 ± 1 h)	Y	Y	Y	Cloudy	2.28	122.62 ± 45.62	13.00
	10/26/2013-10/27/2013, (23 ± 1 h)	Y	Y	Y	Cloudy	2.95	89.31 ± 21.79	12.50
	10/27/2013-10/28/2013, (23 ± 1 h)	Y	Y	Y	Shower	3.67	51.79 ± 15.95	12.00
	10/28/2013-10/29/2013, (23 ± 1 h)	N	N	Y	Shower	4.92	26.67	13.00
	10/29/2013-10/30/2013, (23 ± 1 h)	N	N	Y	Shower	4.78	54.08	14.00
Winter	1/9/2014-1/10/2014, (23 ± 1 h)	Y	Y	Y	Clear	5.50	101.42 ± 46.56	8.50
	1/10/2014-1/11/2014, (23 ± 1 h)	Y	Y	Y	Clear	8.09	131.08 ± 74.13	11.50
	1/11/2014-1/12/2014, (23 ± 1 h)	Y	Y	Y	Clear	6.88	127.56 ± 69.12	10.50
	1/12/2014-1/13/2014, (23 ± 1 h)	Y	Y	Y	Clear	4.83	67.16 ± 1.72	9.50
	1/13/2014-1/14/2014, (23 ± 1 h)	Y	Y	Y	Sleet	4.25	152.41 ± 40.89	4.00
	1/14/2014-1/15/2014, (23 ± 1 h)	N	N	Y	Cloudy	4.47	132.29	7.00
	1/15/2014-1/16/2014, (23 ± 1 h)	N	N	Y	Cloudy	6.93	113.70	7.50

WM: West Mountain, Clean reference sampling site; JDM: Jinding Mountain, Industrial sampling site; DR: Dongfeng East Road, Traffic-intensive sampling site; m·s^−1^: Speed unit, expressed in meters per second; μg·m^−3^: Concentration unit, indicating the number of micrograms per cubic meter; SD: Standard deviation; *n*: Number of samples; °C: Degree Celsius; h: Hour; Y: Sampling; N: No sampling.

**Table 2:** The exposure parameter values.

Parameter	Physical Significance	Value			Unit
		Children	Men	Women	
*EF*	Exposure relative frequency	365	365	365	d·a^−1^
*ED*	Exposure duration	6	30	30	a
*CF*	Conversion coefficient	10^−6^	10^−6^	10^−6^	kg·mg^−1^
*IRi*	Inhalation rate	5	15.2	11.3	m^3^·d^−1^
*BW*	Average body rate	15	62.7	54.4	kg
*AT*	Averaging time (non-carcinogens)	2190	10,950	10,950	d
	Averaging time (carcinogens)	25,500	25,500	25,500	d

d: Day; a: Year: kg: Kilogram; mg: Milligram; m: Meter.

**Table 3:** Response parameters of elements entering through the respiratory system [49,50,51].

Element	Risk	*Rfd* (mg·(kg·d)^−1^)	*SF* ((kg·d)·mg^−1^)
V	Non-carcinogenic	7 × 10^−3^	-
Mn	Non-carcinogenic	3 × 10^−4^	-
Cu	Non-carcinogenic	1.43 × 10^−2^	-
Zn	Non-carcinogenic	0.3	-
Pb	Non-carcinogenic	3.5 × 10^−3^	-
Cr	Carcinogenic	-	42
Co	Carcinogenic	-	32
Ni	Carcinogenic	-	0.84
As	Carcinogenic	-	20.07
Cd	Carcinogenic	-	8.4

V: Vanadium; Cr: Chromium; Mn: Manganese; Co: Cobalt; Ni: Nickel; Cu: Copper; Zn: Zinc; As: Arsenic; Cd: Cadmium; Pb: Lead; Rfd: The reference dose of each heavy metal; SF: The carcinogenic slope factor; kg: Kilogram; mg: Milligram; d: Day; “-” represents no data.

**Table 4:** Concentrations of heavy metals in PM_2.5_ in Kunming compared with other cities (Unit: ng·m^−3^).

Cities	Altitude (m)	Sampling Sites	Year	Season	PM_2.5_ (μg·m^−3^)	V	Cr	Mn	Co	Ni	Cu	Zn	As	Cd	Pb	Ref.
Kunming (Mean±SD, *n* = 68)	1891	urban	2013~2014	Four seasons	130.45 ± 86.35	40.30 ± 92.08	29.10 ± 32.21	155.79 ± 68.71	2.20 ± 2.06	20.50 ± 14.76	78.80 ± 127.37	327.00 ± 233.78	26.76 ± 21.92	6.00 ± 4.42	281.50 ± 225.87	This study
Beijing	43.5	urban	2014	Four seasons	126.00	-	30.00	70.00	-	40.00	200.00	310.00	10.00	-	150.00	[54]
Nanjing	8.9	urban	2013~2014	Summer	97.80 ± 40.50	9.88	13.20	-	-	9.30	24.70	247.00	-	-	90.90	[55]
Chengdu	505.9	urban	2014~2015	Four seasons	-	1.90	5.60	33.80	-	2.10	18.70	238.00	10.80	-	55.40	[56]
Chongqing	259.1	urban	2014~2015	Four seasons	-	-	11.10	37.70	-	4.20	11.30	113.00	-	-	50.40	[56]
Guangzhou	6.6	urban	2014	Four seasons	48.00 ± 22.00	9.00	9.00	34.00	-	4.00	37.00	225.00	-	-	77.00	[57]
Lanzhou	1520	urban	2014	Four seasons	88.90 ± 52.00	-	-	120.00	-	33.80	106.00	237.00	10.50	-	491.00	[60]
Xi’an	396.9	urban	2008~2009	Summer, winter	-	-	152.00	95.00	-	19.00	41.00	1775.00	127.00	8.00	408.00	[53]
Barcelona	20	urban	2001	Four seasons	-	-	6.00	14.00	-	-	32.00	160.00	-	-	120.00	[58]
Istanbul	7	urban	2010	Summer	40.50 ± 13.70	2.54	121.70	42.10	0.52	-	19.60	384.70	-	-	-	[59]

SD: Standard deviation; *n*: Number of samples; μg·m^−3^: Concentration unit, indicating the number of micrograms per cubic meter; ng·m^−3^: Concentration unit, indicating the number of nanograms per cubic meter; V: Vanadium; Cr: Chromium; Mn: Manganese; Co: Cobalt; Ni: Nickel; Cu: Copper; Zn: Zinc; As: Arsenic; Cd: Cadmium; Pb: Lead; Ref.: References; “-” represents no data.

**Table 5:** Correlation coefficient of heavy metals in PM_2.5_ of Kunming (*n* = 68).

	V	Cr	Mn	Co	Ni	Cu	Zn	As	Cd	Pb
V	1									
Cr	0.267	1								
Mn	0.305	0.915 **	1							
Co	−0.058	0.452	0.198	1						
Ni	0.881 **	0.231	0.309	−0.216	1					
Cu	0.821 **	0.561	0.598 *	−0.152	0.870 **	1				
Zn	0.042	−0.019	0.113	−0.183	0.233	0.218	1			
As	−0.090	−0.144	−0.065	−0.191	−0.008	−0.118	−0.012	1		
Cd	0.366	−0.194	−0.162	−0.206	0.596 *	0.316	0.295	0.213	1	
Pb	0.791 **	0.565	0.565	0.213	0.777 **	0.828 **	0.153	0.151	0.394	1

*n*: Number of samples; V: Vanadium; Cr: Chromium; Mn: Manganese; Co: Cobalt; Ni: Nickel; Cu: Copper; Zn: Zinc; As: Arsenic; Cd: Cadmium; Pb: Lead; * Showed significant correlation at 0.05 level (double side); ** Showed significant correlation at 0.01 level (double side).

**Table 6:** PCA analysis of heavy metal elements in PM_2.5_ of Kunming (*n* = 68).

	Cr	Mn	Ni	Cu	Zn	As	Cd	Pb	Variance (%)	Cumulative (%)
Factor 1	0.110	0.018	0.892	0.862	0.724	0.225	0.912	0.880	49.43	49.43
Factor 2	0.735	0.864	−0.129	0.313	0.529	−0.032	−0.089	0.253	18.73	68.16
Factor 3	−0.019	−0.019	−0.097	−0.038	−0.007	0.995	0.157	0.047	12.61	80.77

*n*: Number of samples; Cr: Chromium; Mn: Manganese; Co: Cobalt; Ni: Nickel; Cu: Copper; Zn: Zinc; As: Arsenic; Cd: Cadmium; Pb: Lead; %: Percent sign.

**Table 7:** Non-carcinogenic risk of heavy metals in PM_2.5_ in different groups in Kunming.

Elements	DR			JDM			WM		
	Children	Men	Women	Children	Men	Women	Children	Men	Women
V	5.46 × 10^−4^	3.97 × 10^−4^	3.4 × 10^−4^	9.77 × 10^−4^	7.11 × 10^−4^	6.09 × 10^−4^	3.89 × 10^−4^	2.83 × 10^−4^	2.43 × 10^−4^
Mn	1.72 × 10^−1^	1.25 × 10^−1^	1.07 × 10^−1^	2.11 × 10^−1^	1.53 × 10^−1^	1.31 × 10^−1^	1.37 × 10^−1^	9.93 × 10^−2^	8.51 × 10^−2^
Cu	8.2 × 10^−4^	5.96 × 10^−4^	5.11 × 10^−4^	4.78 × 10^−3^	3.47 × 10^−3^	2.98 × 10^−3^	5.58 × 10^−4^	4.06 × 10^−4^	3.48 × 10^−4^
Zn	3.12 × 10^−4^	2.27 × 10^−4^	1.94 × 10^−4^	6.38 × 10^−4^	4.64 × 10^−4^	3.97 × 10^−4^	1.79 × 10^−4^	1.3 × 10^−4^	1.11 × 10^−4^
Pb	2.06 × 10^−2^	1.5 × 10^−2^	1.29 × 10^−2^	4 × 10^−2^	2.91 × 10^−2^	2.49 × 10^−2^	2.31 × 10^−2^	1.68 × 10^−2^	1.44 × 10^−2^
Sum	1.94 × 10^−1^	1.41 × 10^−1^	1.21 × 10^−1^	2.57 × 10^−1^	1.87 × 10^−1^	1.6 × 10^−1^	1.61 × 10^−1^	1.17 × 10^−1^	1 × 10^−1^

WM: West Mountain, Clean reference sampling site; JDM: Jinding Mountain, Industrial sampling site; DR: Dongfeng East Road, Traffic-intensive sampling site; V: Vanadium; Mn: Manganese; Cu: Copper; Zn: Zinc; Cd: Cadmium; Pb: Lead; Sum: Summation.

**Table 8:** Carcinogenic risk of heavy metals in PM_2.5_ posed to different groups in Kunming.

Elements	DR			JDM			WM		
	Children	Men	Women	Children	Men	Women	Children	Men	Women
Cr	4.5 × 10^−5^	1.64 × 10^−4^	1.4 × 10^−4^	2.61 × 10^−5^	9.47 × 10^−5^	8.12 × 10^−5^	2.69 × 10^−5^	9.78 × 10^−5^	8.38 × 10^−5^
Co	1.49 × 10^−6^	5.42 × 10^−6^	4.65 × 10^−6^	3.61 × 10^−5^	1.31 × 10^−5^	1.23 × 10^−5^	1.14 × 10^−6^	4.16 × 10^−6^	3.57 × 10^−6^
Ni	4.09 × 10^−7^	1.49 × 10^−6^	1.27 × 10^−6^	7.84 × 10^−7^	2.85 × 10^−6^	2.44 × 10^−6^	2.82 × 10^−7^	1.03 × 10^−6^	8.78 × 10^−7^
As	9.06 × 10^−6^	3.29 × 10^−5^	2.82 × 10^−5^	2.21 × 10^−5^	8.03 × 10^−5^	6.88 × 10^−5^	1.49 × 10^−5^	5.41 × 10^−5^	4.64 × 10^−5^
Cd	9.75 × 10^−7^	3.55 × 10^−6^	3.04 × 10^−6^	1.97 × 10^−6^	7.18 × 10^−6^	6.15 × 10^−6^	7.7 × 10^−7^	2.8 × 10^−6^	2.4 × 10^−6^
Sum	5.69 × 10^−5^	2.07 × 10^−4^	1.77 × 10^−4^	5.45 × 10^−5^	1.98 × 10^−4^	1.7 × 10^−4^	4.4 × 10^−5^	1.6 × 10^−4^	1.37 × 10^−4^

WM: West Mountain, Clean reference sampling site; JDM: Jinding Mountain, Industrial sampling site; DR: Dongfeng East Road, Traffic-intensive sampling site; Cr: Chromium; Co: Cobalt; Ni: Nickel; As: Arsenic; Cd: Cadmium; Sum: Summation.

## Data Availability

The data used in this paper can be provided by Jianwu Shi (shijianwu@kust.edu.cn).

## Nomenclature

PM_2.5_	Particulate matter with aerodynamic equivalent diameter less than or equal to 2.5 microns in ambient air
V	Vanadium
Cr	Chromium
Mn	Manganese
Co	Cobalt
Ni	Nickel
Cu	Copper
Zn	Zinc
As	Arsenic
Cd	Cadmium
Pb	Lead
DR	Dongfeng East Road sampling site
JDM	Jinding mountain sampling site
WM	West mountain sampling site
SD	Standard deviation
ICP-MS	Inductively coupled plasma mass spectrometry
AMSL	Above mean sea level
CRM	Certified reference materials
EF	Enrichment factor
PCA	Principal component analysis
EPA	U.S Environmental Protection Agency
HRA	Health risk assessment
IRIS	Integrated Risk Information System
IARC	International Agency for Research on Cancer
NOAA	National Oceanic and Atmospheric Administration
ARL	Air Resources Laboratory
BOM	Bureau of Meteorology
NCEP	National Centers for Environmental Prediction
GDAS	Global Data Assimilation System
